# Supplementary material for: Lipocalin-2 is an anorexigenic signal in primates
Source: eLife. 2020 Nov 24;9:e58949. doi: 10.7554/eLife.58949 (PMC7685704; doi:10.7554/eLife.58949)
Supplement: Supplementary file 1. — (A) Characteristics of participants of the 1st and 2nd study. (B) Characteristics of study participants of the 3rd study. (C) Characteristics of participants of the 4th study. [file elife-58949-supp1.docx]

| **Supplementary file 1A. Characteristics of participants of the 1st and 2nd study.** | | |
| --- | --- | --- |
|  | Study 1  (CUMC 1) | Study 2  (INSERM) |
| Number | 11 | 9 |
| Gender | Females | Females |
| Age (years) | 27.9 ± 0.7 | 26.1 ± 1.0 |
| BMI (kg/m^2^) | 21.8 ± 0.6 | 20.8 ± 0.5 |
| Fasting glucose (mg/dL) | 79.9 ± 1.3 | - |
| Fasting LCN2 (ng/mL) | 38.5 ± 7.6 | 74.4 ± 8.8 |
| Peak LCN2 (ng/mL) | 44.6 ± 7.8 (‡) | 114.6 ± 16.5 (*) |
| Change from baseline | 16% | 54% |
| Fasting GLP-1 (ng/mL) | 51.9 ± 3.5 | 11.9 ± 2.0 |
| Peak GLP-1 (ng/mL) | 85.5 ± 5.5 (*) | 18.0 ± 2.0 (*) |
| Change from baseline | 65% | 90% |
| Major GLP-1 change at (min) | 45 | 60 |
| Major LCN2 change at (min) | 45 | 60 |
| Fasting insulin (mIU/mL) | 1.6 ± 0.3 | 8.9 ± 1.9 |
| Peak insulin (mIU/mL) | 9.2 ± 2.6 (*) | 65.4 ± 16.4 (*) |
| Change from baseline | 473% | 659% |
| Major insulin change at (min) | 30 | 60 |
| Contains raw (i.e. non-transformed data) based on which % change of postprandial concentrations was calculated. Values represent mean ± SEM. * indicates P<0.05 and ‡ indicates P<0.06 versus fasting state for each protein with two-tailed Student’s t-, Wilcoxon or Mann Whitney tests. *BMI = Body Mass Index, GLP-1 = Glucagon-like peptide 1, LCN2 = Lipocalin-2, IU=International Units.* | | |

| **Supplementary file 1B. Characteristics of study participants of the 3^rd^ study.** | | | |
| --- | --- | --- | --- |
|  | *Study 3*  *(Rutgers)* | | |
| *Number* | 47 (R:25, NR 22) | 28 (R: 15, NR: 13) | 19 (R: 10, NR:9) |
| *Gender* | Mixed | Females | Males |
| *Age (years)* | R: 32.1 ± 2.4  NR: 32.8 ± 2.6 | R: 36.4 ± 3.5  NR: 35.6 ± 3.9 | R: 25.7 ± 2.0  NR: 28.7 ± 2.7 |
| *BMI (kg/m^2^)* | R: 28.2 ± 0.6  NR: 29.2 ± 0.7 | R: 27.9 ± 0.7  NR: 30.0 ± 1.1 | R: 28.7 ± 1.3  NR: 28.1 ± 0.9 |
| *% Body Fat* | R: 26.4 ± 1.4  NR: 28.2 ± 1.4 | R: 29.4 ± 1.7  NR: 32.1 ± 1.6 | R: 22.1 ± 2.0  NR: 22.6 ± 1.8 |
| *WC (cm)* | R: 94.6 ± 2.0  NR: 102.4 ± 3.4(†) | R: 92.3 ± 2.5  NR: 104.0 ± 5.4(*) | R: 98.2 ± 3.7  NR: 100.1 ± 4.9 |
| *SBP (mm Hg)* | R: 110.0 ± 2.8  NR: 107.9 ± 3.5 | R: 110.9 ± 3.7  NR: 105.0 ± 3.5 | R: 108.6 ± 5.2  NR: 112.0 ± 7.9 |
| *DBP (mm Hg)* | R: 81.8 ± 2.0  NR: 83.6 ± 2.4 | R: 79.1 ± 2.1  NR: 83.3 ± 3.8 | R: 86.0 ± 4.1  NR: 84.0 ± 3.1 |
| *Fasting Glucose (mg/dL)* | R: 92.2 ± 1.4  NR: 94.5 ± 1.9 | R: 93.5 ± 1.9  NR: 95.6 ± 3.2 | R: 90.3 ± 2.4  NR: 92.8 ± 2.4 |
| *Fasting Lipocalin-2 (ng/mL)* | R: 34.7 ± 4.1  NR: 37.6 ± 2.8(†) | R: 33.0 ± 3.6  NR: 39.4 ± 4.2 | R: 37.1 ± 8.9  NR: 35.1 ± 3.1 |
| *Peak Lipocalin-2 (ng/mL)* | R: 38.8 ± 4.9  NR: 30.5 ± 2.2 | R: 36.3 ± 4.2  NR: 30.7 ± 3.5 | R: 42.7 ± 10.4  NR: 29.7 ± 3.0 |
| *Change from baseline* | R: +12%  NR: -19% | R: +10%  NR: -22% | R: +15%  NR: -16% |
| *Major LCN2 change at (min)* | R: 60  NR: 60 | R: 60  NR: 60 | R: 60  NR: 90 |
| *Fasting GLP-1 (ng/mL)* | R: 81.3 ± 6.1  NR: 75.1 ± 7.1 | R: 73.8 ± 7.4  NR: 69.0 ± 8.6 | R: 93.3 ± 9.9  NR: 81.7 ± 11.6 |
| *Peak GLP-1 (ng/mL)* | R: 115.0 ± 7.1  NR: 97.0 ± 7.6 | R: 113.0 ± 8.5  NR: 104.2 ± 13.2 | R: 114.7 ± 12.3  NR: 96.3 ± 11.6 |
| *Change from baseline* | R: +41%  NR:+29% | R: +53%  NR: +51% | R: +23%  NR: +18% |
| *Major GLP-1 change at (min)* | R: 90  NR: 120 | R: 90  NR: 90 | R: 90  NR: 120 |
| *Fasting insulin (mIU/mL)* | R: 17.2 ± 1.3  NR: 15.9 ± 1.4 | R: 17.9 ± 2.0  NR: 16.3 ± 2.0 | R: 16.1 ± 1.4  NR: 15.3 ± 2.1 |
| *Peak insulin (mIU/mL)* | R: 81.7 ± 7.5  NR: 96.1 ± 14.6 | R: 86.6 ± 10.8  NR: 107.2 ± 16.1 | R: 74.4 ± 9.9  NR: 83.9 ± 25.5 |
| *Change from baseline* | R: +375%  NR:+505% | R: +384%  NR: +556% | R: +362%  NR: +447% |
| *Major insulin change at (min)* | R: 30  NR: 30 | R: 30  NR: 30 | R: 30  NR: 30 |
| Contains raw (i.e. non-transformed data) based on which % change of postprandial concentration from baseline was calculated. Values represent mean ± SEM. * indicates P<0.05, and † indicates p<0.1 of R versus NR in each cohort with two-tailed unpaired Student’s t- or Mann-Whitney tests*. R = responders (elevated LCN2 levels in multiple timepoints after the meal), NR = non-responders (reduced LCN2 levels after the meal challenge), BMI = Body Mass Index, WC = waist circumference, SBP = systolic blood pressure, DBP = diastolic blood pressure, LCN2 = Lipocalin-2, GLP-1 = Glucagon-like peptide 1, IU=international units.* | | | |

| **Supplementary file 1C. Characteristics of participants of the 4^th^ study.** | | |
| --- | --- | --- |
|  | *Study 4*  *(CUMC 2)* | |
|  | Pre-RYGB | Post-RYGB |
| *Number of subjects* | 12 | 12 |
| *Gender of subjects* | 11 Females – 1 Male | 11 Females – 1 Male |
| *Age (years)* | 35.3 ± 3.0 | 36.4 ± 3.0 |
| *weight (kg)* | 128.9 ± 6.5 | 80.4 ± 5.2 (*) |
| *BMI (kg/m^2^)* | 47.4 ± 1.9 | 29.6 ± 1.8 (*) |
| *Fasting Glucose (mg/dL)* | 98.2 ± 3.8 | 86.2 ± 2.7 (*) |
| *Fasting LCN2 (ng/mL)* | 61.4 ± 8.0 | 47.1 ± 4.8 (†) |
| *Peak LCN2 (ng/mL)* | 49.5 ± 6.6 | 75.0± 20.1 (*) |
| *LCN2 Change from baseline* | -16% | +59% |
| *Major LCN2 change at (min)* | 90 | 90 |
| *Fasting GLP-1 (ng/mL)* | 7.4 ± 0.9 | 9.0 ± 1.3 |
| *Peak GLP-1 (ng/mL)* | 16.8 ± 2.3 | 92.2 ± 18.6 (*) |
| *GLP-1 Change from baseline* | 140% | 1176% |
| *Major GLP-1 change at (min)* | 30 | 45 |
| *Fasting insulin (uIU/mL)* | 22.4 ± 1.9 | 5.7 ± 0.9(*) |
| *Peak insulin (uIU/mL)* | 147.0 ± 30.7 | 267.8 ± 61.6(‡) |
| *Insulin Change from baseline* | +547% | +5935% |
| *Major insulin change at (min)* | 30 | 60 |

Contains raw (i.e. non-transformed data) based on which % change of postprandial LCN2 concentration was calculated. LCN2 Values represent mean ± SEM. * indicates P<0.05, ‡ indicates P<0.06, † indicates P<0.1 of Post-RYGB versus Pre-RYGB with two-tailed paired Student’s t-tests or Wilcoxon tests. RYGB = Roux-en-Y Gastric Bypass, BMI = Body Mass Index, LCN2 = Lipocalin-2, GLP-1 = Glucagon-like peptide 1, IU = International Units.
